# Left atrial fibrosis in atrial fibrillation: Mechanisms, clinical evaluation and management

**DOI:** 10.1111/jcmm.16350

**Published:** 2021-02-11

**Authors:** Jin Ma, Qiuxiong Chen, Shiyu Ma

**Affiliations:** ^1^ The Second Affiliated Hospital of Guangzhou University of Chinese Medicine Guangdong Provincial Hospital of Chinese Medicine Guangzhou China

**Keywords:** arrhythmia, atrium, cardiovascular imaging, electrophysiology, fibrosis

## Abstract

Atrial fibrillation (AF), the commonest arrhythmia, shows associations with various disease conditions. Mounting evidence indicates that atrial fibrosis is an important part of the arrhythmogenic substrate, with an essential function in the generation of conduction abnormalities that underlie the transition from paroxysmal to persistent AF, which in turn contributes to AF perpetuation. Left atrial (LA) fibrosis is considered a possible major factor and predictor in AF treatment. The present review provides insights into LA fibrosis’ association with AF. The information is focused on clinical aspects and mechanisms, clinical evaluating methods that evaluate fibrosis changes and examining possible options for the prevention of atrial fibrosis.

## 1 INTRODUCTION

Atrial fibrillation (AF) represents the most commonly described cardiac arrhythmia; it presently affects more than 33 million people globally, a number expected to rise by more than twofold in the coming four decades. AF elevates the risks of stroke, myocardial infarction, heart failure, dementia and chronic kidney disease (CKD), and constitutes an important risk factor for mortality. The incidence of AF increases rapidly with age, and the majority of patients are aged >65 years. The increasing prevalence of AF will likely result in increased financial burden on patients/families and the society. Consequently, AF is considered a threat to human health worldwide.

Atrial fibrillation has complex mechanisms, which have been intensively explored in the past. It is associated with various ailments, including hypertension, diabetes, coronary artery disease and ageing. Triggers and substrates that mediate AF pathogenesis are likely interconnected. Atrial fibrosis is increasingly considered an important part of vulnerable substrates, with an essential function in the induction of left atrial (LA) conduction abnormalities underlying the transition from paroxysmal to persistent AF and contributing to disease continuation. LA fibrosis, an important event in AF pathogenesis, represents a critical risk factor for adverse outcome in AF cases. However, whether the increasing recognization of atrial fibrosis in AF pathogenesis translates into improved efficiencies of catheter ablation and antiarrhythmic treatments is undefined. This review aimed to summarize clinical and mechanisms studies of LA fibrosis as a parameter that could be applied for the development, evaluation and treatment of AF.

## 2 CLINICAL STUDIES DEMONSTRATE THAT LA FIBROSIS IS CLOSELY ASSOCIATED WITH AF

### 2.1 LA fibrosis strongly predicts AF recurrence

Fibrotic myocardial tissue comprises disorderly arranged myocytes and collagen, with expanded extracellular space in comparison with non‐diseased myocardium. With the development of non‐invasive assessment techniques for myocardial fibrosis, the degree of LA fibrosis constitutes a good predictive factor of AF recurrence and related disease risk. Previous studies using imaging assessment to detect structural alteration of atrial tissue have helped further understand the pathophysiological mechanisms of AF and disease progression. Meanwhile, LA fibrosis is independently associated with arrhythmia recurrence post‐AF ablation.^1^ According to the degree of fibrosis assessed by delayed enhancement MRI, four stages could be defined, including stages 1 (<10% of the atrial wall), 2 (between 10% and 20%), 3 (20%‐30%) and 4 (≥30%). A retrospective analysis of 426 patients followed up for 1 year demonstrated that recurrent arrhythmias are strongly correlated with the degree of LA fibrosis, with stages I, II, III and IV having 21%, 29.3%, 33.8% and 71.4% of recurrent arrhythmias cases, respectively.^2^ Another retrospective assessment of 308 cases with a 5‐year follow‐up revealed that the degree of LA fibrosis is tightly associated with long‐term outcome of AF ablation. In the latter study, patients with more advanced atrial fibrosis were more likely to experience recurrent AF (hazard ratio for stage IV vs stage I, 2.73; 95% confidence interval [CI]: 1.57‐4.75) and to undergo a subsequent ablation (proportional odds ratio for stage IV vs stage I, 5.19; 95% CI: 2.12‐12.69).^3^ LA fibrosis degree is a strong predictor of the long‐term maintenance of normal sinus rhythm following AF ablation.

### 2.2 Association of LA appendage (LAA) thrombosis with fibrosis and AF

AF is the end‐point of a disease characterized by morphological and structural changes in the atrium. These changes can enhance the probability of thrombus generation. The majority of LA thrombi are found in the LAA. In a transoesophageal echocardiography (TEE) trial of 317 AF cases with a recently observed embolic event, approximately 20% individuals exhibited thrombi in the LAA.^4^ A recently reported study revealed LAA fibrosis is related to decreased LAA’s flow rate,^5^ as determined by cardiac magnetic resonance (CMR), indicating that fibrotic alterations of the LAA are related to blood stagnation, thrombus generation and stroke occurrence.^6^ Because of stasis and coagulation induction in the fibrillating atrium, there is elevated risk of thromboembolism, especially ischaemic stroke, with a total stroke risk of 5% yearly.^7^, ^8^ LA fibrosis, an important factor in structural atrial remodelling, can be used in conjunction with the CHADS2 (congestive heart failure, hypertension, age ≥ 75 years, diabetes mellitus, and prior stroke or transient ischaemic attack) index for anticoagulation risk stratification.^9^ AF cases with previous ischaemic stroke show remarkably elevated degree of LA fibrosis in delayed enhancement MRI. Daccarett and collaborators reported substantially increased fibrosis incidence in individuals with previous stroke (24.4%) in comparison with counterparts without this condition (16.2%), with age, AF type and warfarin use not affecting this association. A further study demonstrated that stroke is associated with LA fibrosis in individuals with embolic stroke of undetermined source (ESUS) who exhibited a higher atrial fibrosis incidence compared with control subjects; similar incidence of fibrosis was also noted in comparison with AF (*P* *>* .05).^10^ These authors concluded that fibrosis has an association with ESUS following stratification for stroke risk factors. Fibrosis causes cardioembolic stroke in an AF‐independent manner. Jointly, the above findings suggest that elevated LA fibrosis degree increases the risk of thromboembolism. In the past years, left atrial appendage closure (LAAC) and oral anticoagulation have been observed in prospective randomized studies, indicating optimal efficacy and safety in the prevention of stroke and systemic embolism associated with AF.^11^

### 2.3 The causal relationship between atrial fibrosis and AF

Whether fibrosis of atrial myocardium is a cause or a consequence of AF in cardiovascular disease cases is currently undefined. Historically, atrial fibrotic remodelling has been considered to result from AF, in turn perpetuating AF. However, electro‐anatomical mapping and non‐invasive heart imaging data suggest that fibrosis possibly precedes AF occurrence. Previous studies have demonstrated AF’s association with LA’s electrical, contractile and structural remodelling, which is involved in the persistence of arrhythmias.^12^ In addition, such remodelling process ends with loss of atrial myocytes and elevated collagen amounts, and subsequent LA wall fibrosis. Electrophysiological and imaging findings demonstrate that reduced voltage and fibrotic LA tissue independently predict surgical outcome. Accurately and reliably measuring LA fibrosis might ameliorate decision‐making by clinicians.^13^ Wilson et al^14^ analysed a familial clustering (694 individuals) of AF/fibrosis cases in Utah and overtly demonstrated that heredity contributes to AF/fibrosis. LA fibrosis is considered an independent predictor of composite adverse outcome.

## 3 MECHANISM STUDIES ON THE CORRELATION BETWEEN ATRIAL FIBROSIS AND AF

### 3.1 Left atrial fibrosis begets AF

**FIGURE 1:**
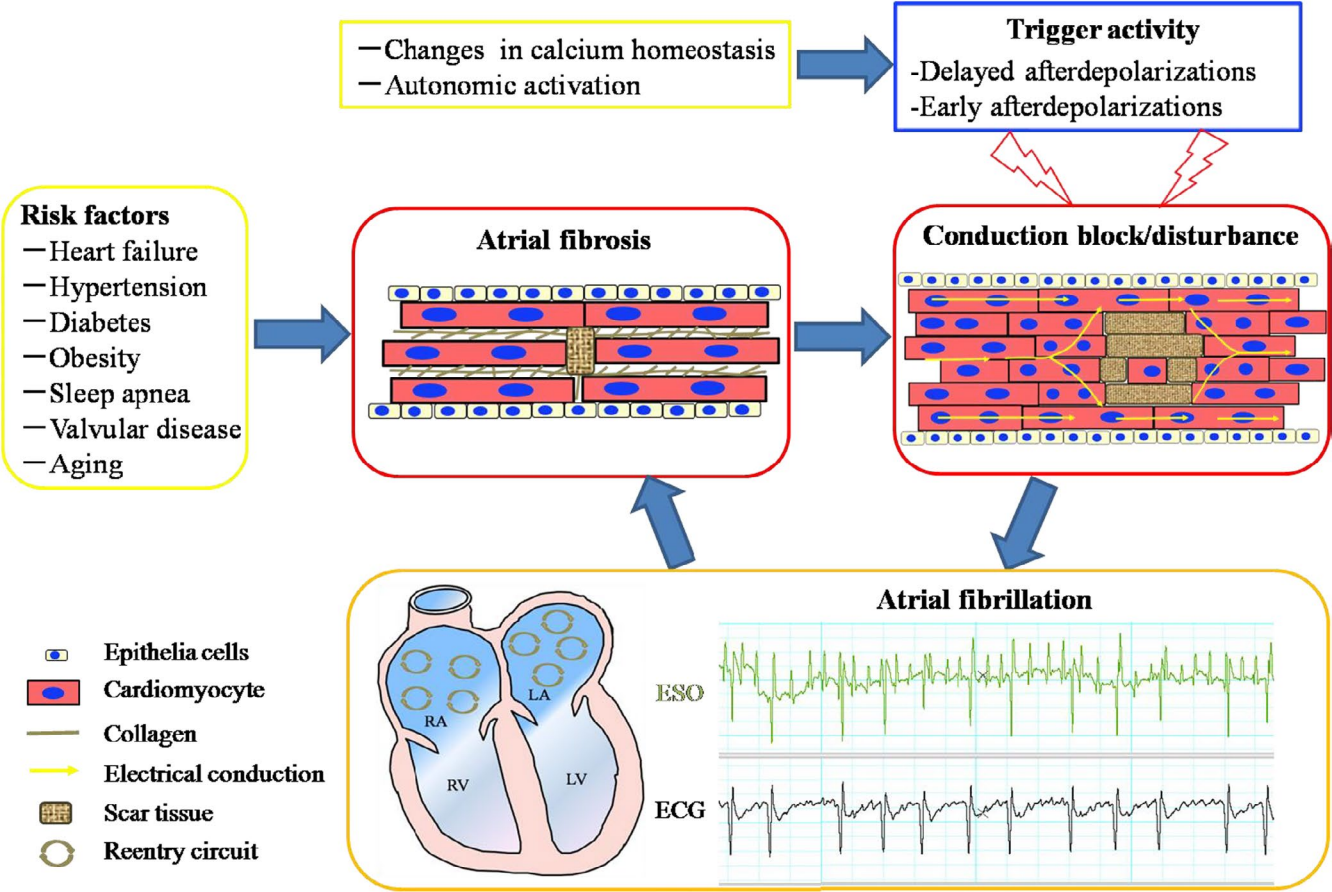
The malignant cycle of atrial fibrosis and AF. The development and maintenance of AF require both a trigger and a susceptible substrate. Triggers are mainly represented by ectopic discharges originating in the PV ostia, due to changes in calcium homeostasis or autonomic activation which can trigger activity involving early afterdepolarizations (EADs) and delayed afterdepolarizations (DADs). These ectopic discharges can initiate AF by premature atrial electrical activation. In addition to this process, an AF substrate is essential to maintain the arrhythmia. Structural remodelling, particularly atrial interstitial fibrosis, is the main arrhythmogenic substrate and leads to local conduction disturbances and block, which increases the risk of re‐entry circuits. AF itself can further perpetuate the progression of atrial fibrosis. ECG, electrocardiogram; ESO, oesophageal electrocardiogram

The exact mechanism by which fibrosis contributes to AF initiation and maintenance remains unknown. AF represents a complex and progressive ailment (Figure 1).^15^ Ectopic (triggered) impact and re‐entry constitute the main arrhythmogenic mechanisms of AF.^16^ Triggers were previously considered to be localized to the pulmonary vein (PV) ostia. However, enhanced fibrosis might generate novel, non–PV‐associated induction sites. Changes in atrial refractoriness, cell calcium homeostasis/handling and autonomic activation are delayed or occur early following depolarization. These processes could participate in induced activity or ectopic focal discharges triggering AF. Every AF event is initiated by a trigger affecting a vulnerable substrate. Increased fibrosis changes the conduction properties of the tissue and causes local conduction changes and conduction blockage. This increases the odds of re‐entry circuits, such that fibrosis is a major contributor to AF maintenance.^17^

Clinically, atrial remodelling generally occurs because of rapidly developing atrial tachyarrhythmia or as a consequence of altered atrial structure following pressure or volume overload. Atrial fibrosis represents an important feature of arrhythmogenic structural remodelling.^18^ Common comorbidities of heart failure include hypertension and heart valve disease that can promote atrial fibrosis for AF development and perpetuation.^19^ Animal experiments have convincingly demonstrated that fibrosis enhances conduction heterogeneity through the left atrium, facilitating circuit re‐entry and conduction disturbance. These processes lead to delays, wave breaks and lines of block in the atrial wall, which might directly help maintain AF.^20^ Olgin et al demonstrated that atrial fibrosis alone can enhance AF inducibility in transgenic mice with TGF‐β1 overexpression. The contribution of LA scars to AF development has been reported for multiple experimental and clinical biopsies.^21^ Atrial fibrosis begets AF and could independently predict AF occurrence.^22^

### 3.2 AF causes left atrial fibrosis

Atrial fibrillation‐associated structural remodelling features tachycardia‐related atrial fibrosis, which has a critical function in AF development and perpetuation.^23^ Biopsies and autopsies in clinical and experimental studies of AF have revealed atrial fibrosis. In larger models, atrial and ventricular tachypacing has been demonstrated to lead to atrial fibrosis, which subsequently increases the odds of AF perpetuation. In previously reported dog and goat rapid pacing AF models, Morillo and colleagues and Schotten and collaborators examined structural and ultrastructural alterations occurring in the atria upon AF. Li et al induced experimental CHF by a 5‐week rapid ventricular pacing (220‐240 bpm), which enhanced and promoted AF perpetuation via interstitial fibrosis interfering with local conduction. The two studies by Li et al and Avitall et al demonstrated different extent of left atrial fibrosis, which was documented with ablated atrioventricular node and AF, whereas ejection fraction for the ventricle at 80 beats/min was similar (12.8 ± 1.9% vs 14.2 ± 5.3%, respectively)^24^; the above findings suggest significant and similar degrees of atrial fibrosis are noted when AF and LV dysfunction are induced either with a rapid ventricular response to AF or with a clinical HF associated with rapid ventricular pacing.

Atrial fibrillation in the absence of ventricular dysfunction also causes atrial fibrosis and elevates susceptibility to AF. He et al^25^ reported that rapid atrial pacing alone induces substantial alterations in cardiac mRNA levels of collagens and fibrogenic factors in rabbit heart, demonstrating that tachycardia in AF might promote atrial remodelling due to atrial fibrosis. MicroRNA‐21–associated Smad7 suppression decreases the feedback down‐regulation of the TGF‐β1/Smad pathway, providing novel insights into the mechanism underlying AF‐induced atrial fibrosis.^25^ In an in vivo study, the medium of rapidly paced cardiomyocytes reduced [(3)H] thymidine uptake‐increased alpha‐SMA protein, collagen‐1 and fibronectin‐1 mRNA levels in atrial fibroblasts, which indicated that rapid‐paced cardiomyocytes release molecules that substantially affect the function of cardiac fibroblasts, triggering myofibroblast activation characterized by up‐regulated fibrosis‐associated genes. The above studies suggested that AF alone directly increases left atrial fibrosis. Conversion to normal sinus rhythm should be timely performed to prevent atrial fibrosis and AF aggravation.

### 3.3 Activated fibroblasts represent major effector cells in heart fibrosis

**FIGURE 2:**
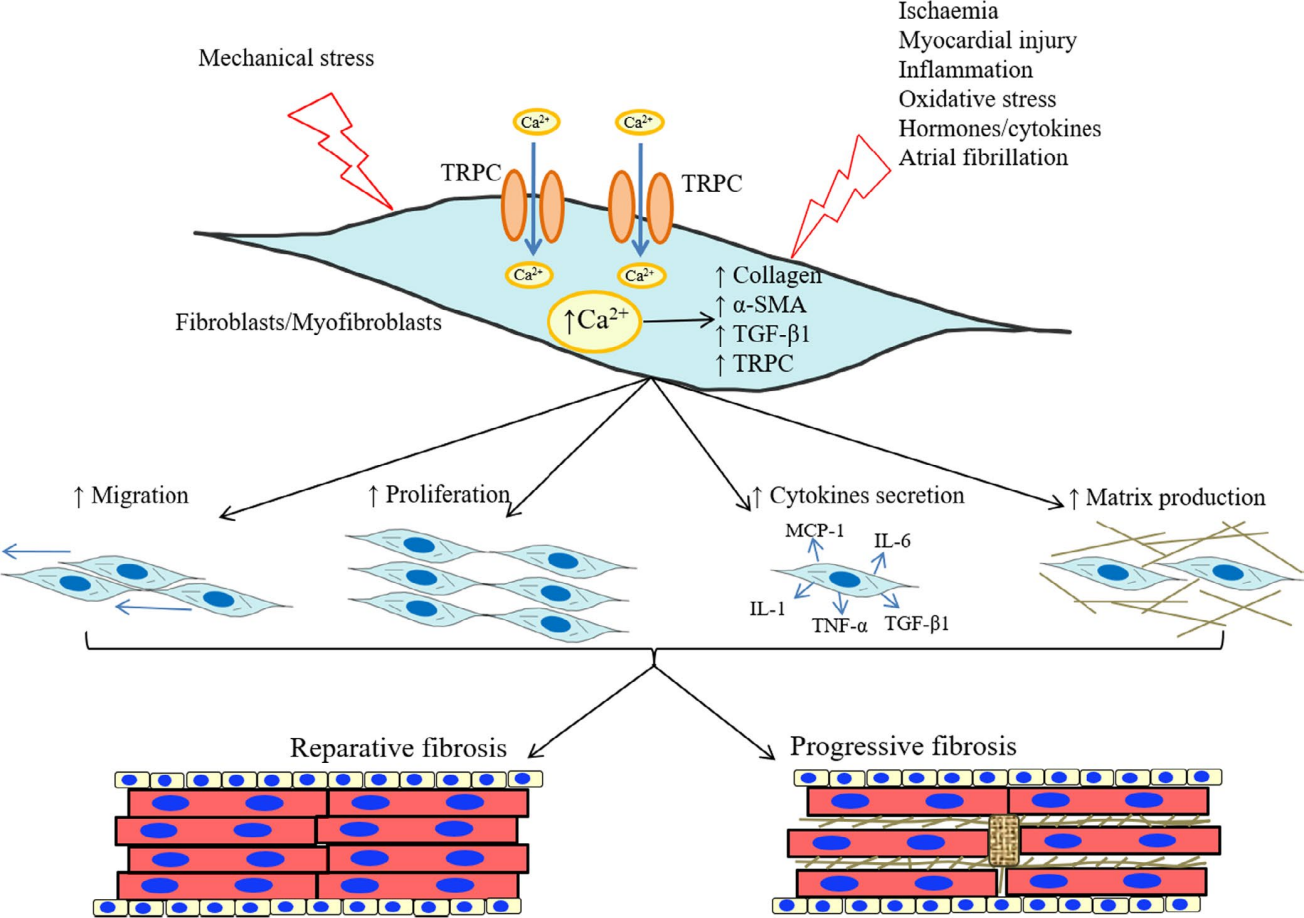
Activated fibroblasts are the main effector cells in heart fibrosis. Under pathological conditions, different stresses stimulate Ca^2+^ entry in fibroblasts via TRP channels (TRPC) that promote a Ca^2+^ concentration increase. Subsequently, the expression levels of specific markers were assessed, such as collagen, α‐SMA, profibrotic cytokine (TGF‐β1) and different isoforms of TRPC, which activate cardiac fibroblasts leading to their migration, proliferation, cytokine secretion and exacerbated extracellular matrix synthesis

Extensive work has revealed the molecular and cellular events occurring during atrial remodelling. The normal heart tissue comprises four main cell groups, including cardiomyocytes, endothelial cells, fibroblasts and smooth muscle cells. The proportions of various cell types are species‐specific. Cardiomyocytes comprise < 50% of all cardiac cells in mammalian heart. Evidence suggests that the cardiac tissue has about 70% non‐myocyte cells. It is important to note that fibroblasts are the most numerous heart cells (40% to >60% of all cells). Activated myofibroblasts constitute the major cells controlling heart fibrosis (Figure 2). Their expansion after myocardial damage occurs mostly via activation of resident interstitial cells. Additional cells such as cardiomyocytes, endothelial cells, pericytes, macrophages, lymphocytes and mast cells might be involved in fibrosis via production of proteases, which contribute to matrix metabolism.^26^ Under normal conditions, fibroblasts are involved in myocardial function, constituting a cell scaffold that maintains an adequate three‐dimensional network needed for normal mechanical function and contributes to generate uniform excitable substrate that remains uninterrupted and can rapidly propagate electrical activation through the myocardium. Pathological conditions trigger fibroblast proliferation and migration, with fibroblasts undergoing phenotypic alterations contributing to differentiation into myofibroblasts, the major extracellular matrix (ECM)–secreting cells.^27^ This differentiation causes functional modifications such as elevated proliferation, enhanced release of signalling factors and promoted ECM accumulation. This fibrotic process fills the tissue, maintaining structural integrity following the death of cardiomyocytes. However, myofibroblast persistence in the damaged area transforms this beneficial response into a deleterious one that causes progressive fibrosis. Excess ECM synthesized by fibroblasts decreases cardiomyocyte‐bundle continuity, resulting in local conduction disturbance and recurrent arrhythmias.

In the heart tissue, fibroblasts can directly interact with myocytes via paracrine mechanisms. Following stimulation, myofibroblasts secrete cytokines, including TGF‐β1 and TNF‐α.^28^ This affects fibroblasts differentiation into myofibroblasts, the activation of the fibrotic pathway and ECM production.^28^ Cytokine secretion further promotes re‐entry through direct electric interactions with cardiomyocytes by reducing conduction velocity in the infarcted heart. Cardiac fibroblasts produce multiple ion channels, especially voltage‐gated K^+^ channels and non‐selective cation channels of the transient receptor potential (TRP) family, which control fibroblast function, with TRP channels enabling Ca^2+^ entry to induce fibroblast differentiation into secretory myofibroblasts, which in turn synthesize ECM proteins. In addition, fibroblasts interact with cardiomyocytes and profoundly alter their electrical features such as conduction, resting potential, repolarization and excitability. Fibroblasts constitute the major cell group controlling fibrosis and affecting the AF substrate.

### 3.4 Factors controlling cardiac myofibroblast activation

A few factors have been identified that independently induce cardiac fibroblast and other cells (eg endothelial cells, epithelial cells and bone marrow–derived precursors) differentiation into myofibroblast, including mechanical stress, ischaemic damage, inflammation, reactive oxygen species (ROS) and high‐frequency electrical activity (Figure 2).^29^ The changing mechanical environment induces fibroblasts to differentiate. Under increased extracellular matrix stiffness and mechanical strain, cardiac fibroblasts can undergo myofibroblast differentiation.^30^ But the comprehensive mechanisms for mechanoregulation of cardiac myofibroblast differentiation remain elusive. Besides mechanical conditions, the differentiation is triggered by several cytokines, chemokines, neurohumoural factors and vasoactive peptides circulating throughout the injured environment. These chemical signals include profibrotic (TGF‐β), inflammatory cytokines (TNF‐α, IL‐1β and IL‐6) and ROS from NADPH oxidase as well as the angiotensin II (Ang II) and endothelin‐1 (ET‐1). TGF‐β is a vital growth factor that induces differentiation of fibroblasts to myofibroblasts through the activation of Smad2/3‐dependent or Smad2/3‐independent signalling pathways.^31^ Ang II, a neurohumoural factor serving as upstream inducer of TGF‐β signalling for myofibroblast differentiation, has been shown to increase the expression of ET‐1 in cardiac fibroblasts.^32^ ET‐1, a bioactive peptide produced during cardiac injury, appears to function synergistically and downstream of both Ang II and TGF‐β to promote and maintain the myofibroblast phenotype.^31^ After injury, the inflammatory response in the heart may be the primary trigger for phenotype switch. A broad range of inflammatory cytokines, such as TNF‐a and interleukins, affects the fibroblast activation directly.^33^ The chronic release of ROS, linked to the development of left ventricular hypertrophy and heart failure progression, increases TGF‐β–mediated activation of Smad2/3 to indicate a potentially important role for ROS in TGF‐β–stimulated conversion of fibroblast to myofibroblast phenotype.^29^ Mechanical and biochemical cues are often interdependent in cardiac myofibroblast differentiation process.

## 4 CLINICAL EVALUATION FOR ASSESSING ATRIAL FIBROSIS AND AF

### 4.1 Late gadolinium enhancement magnetic resonance imaging (LGE‐MRI)

LGE‐MRI constitutes the gold standard for imaging myocardial fibrosis, which could assess the presence, pattern and size of replacement or focal fibrosis in scarred tissues. The scar border zone is considered a powerful predictor of ventricular arrhythmia risk.^34^ LA fibrosis evaluation is not broadly applied clinically. Gadolinium (Gd), a paramagnetic metal, accumulates in the extracellular space of the myocardium, modifying the magnetic features of water. More specifically, as Gd preferentially locates between cells, and blood vessels are less voluminous in fibrosis, it has prolonged localization to and removal from the extracellular compartment.^35^ Therefore, delayed Gd enhancement in MRI, which involves elevated signal intensities in diseased regions, is related to tissue fibrosis.^36^ Gd assessment in LGE‐MRI could help quantitate ECM volume and represents a surrogate marker of myocardial fibrosis.^3^, ^37^ Although the degree of fibrosis might predict recurrence following ablation, the lack of normal standard values has inspired the development of multiple image acquisition and post‐processing techniques and thresholds for detecting fibrosis, eventually hindering the external validation and reproducibility of this method.^35^ Formulation of reference values for imaging atrial fibrosis can be used for detecting diffuse fibrosis via LGE‐MRI. This might provide novel insights into the role of atrial fibrosis in the pathophysiological mechanisms of various heart diseases.

### 4.2 Speckle tracking echocardiography (STE)

Left atrial strain represents an important STE‐related index analysing chamber function, with highly repeatable measurements of LA impairment by non‐Doppler, angle‐independent assessment. STE was designed to evaluate ventricular function but has been intensely applied for measuring atrial chamber function recently. The speckle pattern (acoustic backscatter from the reflected ultrasonic beam) is followed frame by frame, with the best matching area identified statistically. Displacement of the assessed pattern theoretically follows the myocardial movement, and each alteration between speckles represents a myocardial distortion. Atrial longitudinal strain represents a critical factor for analysing LA function in AF.^38^ Kuppahally et al revealed an inverse association of fibrosis degree assessed by LGE CMR and LA strain, notably in cases of persistent AF in comparison with acute forms.^39^ Recent prospective long‐term follow‐up studies demonstrated that longitudinal strain is a potent and independent predictive factor of future AF occurrence in different cohorts.^40^, ^41^ Longitudinal strain predicts prognosis in AF, independently of cardiovascular risk factors.

### 4.3 Electro‐anatomical voltage mapping (EAM)

In patients undergoing ablation procedures, intracardiac EAM can identify the presence, location and extent of fibrosis in the LA by detection of low‐voltage tissue regions, which are the substrate for tachyarrhythmias. Low‐voltage areas in the LA can indicate increased likelihood of AF recurrence after ablation.^42^ LA voltage areas more than 10% of the total surface area predicts arrhythmia recurrence following pulmonary vein antral isolation for paroxysmal AF.^43^ Several studies have also shown a high level of agreement between regions of fibrosis identified by LGE‐MRI and low‐voltage areas identified by EAM.^44^

### 4.4 Electrocardiogram (ECG)

Electrocardiogram carries important information about electrophysiological properties of the heart, which is an affordable and non‐invasive tool that enables the estimation of atrial conduction properties which correlate with fibrosis. P‐wave characteristics reflect underlying atrial electrophysiology, as well as structure and function. Interatrial block (IAB), defined as prolonged P‐wave duration (>120 ms) on a 12‐lead ECG, reflects delayed conduction between the right and the left atrium.^45^ Atrial fibrosis is considered the major contributor to the underlying pathophysiological mechanism of IAB.^46^ Previous studies have provided good evidence that advanced IAB is associated with increased risk of incident AF,^47^ AF recurrence after catheter ablation, stroke. Advanced IAB in sinus rhythm is independently associated with AF and stroke in the population with structural heart disease and no previous diagnosis of AF.^48^ Clinicians may consider monitoring patients with IAB more closely for the occurrence of AF, especially for advanced subgroups.

### 4.5 Biomarkers of fibrosis

Except for tests that directly visualize atrial fibrosis and remodelling (LGE‐MRI, STE and EAM), circulating biomarkers, including the most well studied (collagen peptides, miRNAs and galectin‐3), may therefore be used as markers of left atrial remodelling. Circulating markers of collagen synthesis (eg propeptide of type III procollagen, PIIINP^49^) and turnover (eg carboxy‐terminal telopeptide of collagen type I,CITP^50^), collagen‐degrading MMPs (eg MMP2^51^) are involved in the process of LA remodelling and potentially might also permit non‐invasive and indirect assessment of LA fibrosis. Some important miRNAs that have been implicated as regulators of atrial fibrosis in AF. Circulating and tissue miRNAs regulate determinants of AF pathophysiology and have emerged as biomarkers of this disease, such as miR21, miR26, miR29b, miR‐30a, miR‐30 and miR‐133. Galectin‐3 involves macrophage and fibroblast recruitment resulting in cell proliferation and collagen accumulation, and it plays a role in the electric atrial activity and structural remodelling. High circulating galectin‐3 concentrations were associated with an increased risk of developing AF.^52^ These biomarkers, along with other tests may have significant ramifications for AF monitoring and treatment, allowing for more accurate disease assessment and estimation of various therapeutic interventions success rate.

## 5 TREATMENT APPROACHES TO REDUCE ATRIAL FIBROSIS

### 5.1 Renin‐angiotensin‐aldosterone system (RAAS) antagonists

The RAAS is progressively considered a critical player in cardiac fibrosis development.^53^ Angiotensin‐converting enzyme inhibitors (ACEIs), angiotensin receptor blockers (ARBs)^54^, ^55^, ^56^, ^57^, ^58^, ^59^ and mineralocorticoid receptor antagonists^60^, ^61^, ^62^, ^63^ can reduce atrial fibrosis progression and AF susceptibility, as demonstrated by multiple experimental studies (Table 1).^64^ Retrospective trials and meta‐analyses of randomized studies demonstrated that administration of ACEIs or ARBs is associated with decreased odds of new‐onset AF in heart failure, hypertension and myocardial infarction patients (Table 2). Routine cardiovascular risk factors, such as age, hypertension and heart failure, are associated with AF development and drugs; therefore, controlling them may improve AF prevention. In addition, treatment with ACEIs/ARBs of these diseases may have been confounded by indications.^64^ Treatment of cardiac fibrosis more likely targets its alleviation rather than reversal. In addition, primary AF prevention by these drugs has better feasibility versus secondary prevention. Therefore, angiotensin axis suppressors are recommended for managing AF exclusively in case of arrhythmia combined with additional ailments, which are themselves involved in myocardial fibrotic remodelling, including arterial hypertension with left ventricular hypertrophy and systolic heart failure. In such conditions, angiotensin inhibitors are not indicated for cases with no overt cardiovascular ailment (eg lone AF).^65^

**TABLE 1:** Summary of RAAS antagonists used in animal studies

Authors (refs)	Year	Drug name	Classification	Disease model	N	Primary results
Li et al^58^	2020	Valsartan	ARBs	Rapid atrial pacing AF rabbits	33	AF inducibility↓, AERP↑, LA diameter↓, LA collagen volume fraction↓, collagen I↓, collagen III↓
Takemoto et al^60^	2017	Eplerenone	Aldosterone inhibitor	Atrially tachypaced remodelling sheep	34	AF inducibility↓, AF wave complexity↓, LA area↓, APD↓, Cav1.2↓, serum P3NP↓, SMA↓, collagen III↓
Kataoka et al^54^	2016	Irbesartan	ARBs	Atrial tachycardia remodelling dogs	15	AF inducibility↓, AF duration↓, AERP↑, fibrosis tissue area↓, TGF‐β1↓, P53↑
Wang et al^59^	2015	Telmisartan	ARBs	Spontaneously hypertensive rats	66	AF inducibility↓, AF duration↓, AERP↑, LA diameter ↓, myocyte size↓, fibrosis tissue area↓, PI3K↑, Akt↑
Zhao et al^57^	2013	Losartan	ARBs	Rapid atrial pacing AF rabbits	30	AF inducibility↓, AF duration↓, fibrosis tissue area↓, collagen I↓, collagen III↓, TGF‐β1↓
Kato et al^56^	2011	Candesartan	ARBs	Diabetes rats	–	Fibrosis tissue area↓, CTGF↓
Li et al^80^	2007	Cilazapril	ACEIs	Rapid atrial pacing AF dogs	20	AF inducibility↓, AF duration↓, intraatrium conduction times↓, LAA volumes↓, LAA ejection fraction↑, LAA maximal forward flow velocity↑, LAA minimal backward flow velocity↑, fibrosis tissue area↓
Sakabe et al^81^	2004	Enalapril	ACEIs	Atrial pacing‐induced AF dogs	24	AF duration↓, AF cycle length↓, P‐wave width↓, fibrosis tissue area↓

Abbreviations: ACEIs, angiotensin‐converting enzyme inhibitors; AERP, atrial refractory period; AF, atrial fibrillation; ARBs, angiotensin receptor blockers; CTGF, connective tissue growth factor; LA, left atrial; LAA, left atrial appendage; P3NP, procollagen‐III N‐terminal propeptide; SMA, smooth muscle actin; TGF‐β, transforming growth factor‐β.

**TABLE 2:** Summary of RAAS antagonists used in the clinic

Authors (refs)	Year	Drug name	Classification	N	Time	Primary results
Huang et al^82^	2018	—	ACEIs/ARBs	18 266	8.13 y	Risk of developing new AF↓
Chaugai et al^83^	2016	–	RAAS blocker	165 387	–	Incidence of AF↓ (49% reduction in systolic HF, 19% reduction in new onset and in 54% reduction in recurrent AF in hypertensive patients)
Zhao et al^84^	2015	–	ACEIs/ARBs	42 892	—	Incidence of AF recurrence and congestive HF↓
Han et al^85^	2013	–	ACEIs/ARBs	13 184	–	Incidence of AF recurrence↓
Goette et al^86^	2012	Olmesartan	ARBs	425	12 mo	No effect on the AF episodes in patients with paroxysmal AF without structural heart disease
Heckbert et al^87^	2009	–	ACEIs/ARBs	2322	–	Risk of incident AF↓ (in hypertensive patients without HF)
Komatsu et al^88^	2008	Enalapril	ACEIs	58	43 ± 18 mo	Incidence of AF recurrence ↓ (in patients with paroxysmal AF)
Healey et al^89^	2005	–	ACEIs/ARBs	56 308	–	Relative risk of AF reduced 28%
Maggioni et al^90^	2005	Valsartan	ARBs	4395	23 mo	Relative risk of AF reduced by 37% in the patients with HF
Wachtell et al^91^	2005	Losartan	ARBs	9193	4.8 ± 1 y	Incidence of new‐onset AF and associated stroke ↓ (in hypertensive patients and patients with electrocardiogram‐documented left ventricular hypertrophy)
L'Allier et al^92^	2004	–	ACEIs	8 000 000	4.5 y	Risk of incident AF↓ (in hypertensive patients)
Vermes et al^93^	2003	Enalapril	ACEIs	391	2.9 ± 1 y	Risk of incident AF↓ (in left ventricular dysfunction patients)

Abbreviations: ACEIs, angiotensin‐converting enzyme inhibitors; ARBs, angiotensin receptor blockers.

### 5.2 Anti‐fibrotic drugs

Several other components of the profibrotic cardiac pathways have been identified, including TGF‐β,^66^, ^67^, ^68^ galectin‐3 inhibitors,^69^ inflammatory mediators,^66^ JAK‐STAT signalling,^70^ miRNAs,^25^ TRP channels,^71^ peroxisome proliferator–activated receptor‐gamma^72^ and Chinese herb.^73^, ^74^ These approaches represent attractive therapeutic targets and include drugs with anti‐fibrotic effects that can reduce atrial interstitial fibrosis in animal experiments (Table 3). Whether specific targets are practical and effective in clinical applications remains to be determined.

**TABLE 3:** Summary of anti‐fibrotic drugs that have been used in animal studies

Authors (refs)	Year	Drug name	Classification	Model	N	Primary results
Lee et al^68^	2006	Pirfenidone	TGF‐β inhibitors	CHF canines	15	AF duration↓, fibrosis tissue area↓, LA CV↑, conduction heterogeneous↓, TGF‐β1↓, JNK↓, MMP‐9↓, TNF‐*α*↓, TIMP‐4↑
Harada et al^71^	2012	Pyrazole‐3	TRP channel blocker	Electrically maintained AF dogs, rat cardiac fibroblasts	48	In vivo: AF burden↓, AERP↑, vimentin↓. In vitro: Ca^2+^ influx↓, proliferation↓, α‐SMA↓
Takemoto et al^69^	2016	GM‐CT‐01	Galectin‐3 inhibitors	Persistent AF sheep, Sheep fibroblasts	24	In vivo: AF inducibility↓, AF burden↓, atrial dominant frequency↓, Rotations↓, singularity points↓, P3NP↓, α‐SMA↓, fibrosis tissue area↓, Smad2/3↓, LA area↓, APD↑. In vitro: migration↓, proliferation↓
Chen et al^70^	2016	S3I‐201	STAT3 inhibitors	Post‐MI mice, canine fibroblasts	25	In vivo: fibrosis tissue area↓, P‐wave width↓. In vitro: STAT3↓, collagen‐1α1↓ and collagen 3α1↓, collagen‐1↓
Liu et al^72^	2017	Pioglitazone	PPAR‐γ activators	Alloxan‐induced diabetic rabbits	96	AF inducibility↓, AF duration↓, LAD↓, IVST↓, LVPWT↓, interatrial conduction time↓, APD↓, fibrosis tissue area↓, p‐ERK↓, TGFβ1↓, TLR4↓, NF‐κB p50↓, HSP70↓
Ma et al^74^	2018	Matrine	Chinese herb	Post‐MI rats	45	In vivo: AF inducibility↓, AF duration↓, LA CV↑, conduction heterogeneous↓, fibrosis tissue area↓, collagen Ⅰ↓, collagen Ⅲ↓, TGFβ1↓, MMP‐9↓, TIMP‐1↓. In vitro: *α*‐SMA↓, proliferation↓, migration↓, secretion↓
Qiu et al^66^	2018	Salvianolate	Chinese herb	Post‐MI rats	63	AF inducibility↓, AF duration↓, P‐Wave width↓, LA diameter ↓, fibrosis tissue area↓, TGF‐β1↓, Smad2/3↓
Ma et al^73^	2019	Tongguan capsule‐derived herb	Chinese herb	Post‐MI rats, cardiac fibroblasts	60	In vivo: AF inducibility↓, AF duration↓, LA CV↑, conduction heterogeneous↓, fibrosis tissue area↓, collagen Ⅰ↓, collagen Ⅲ↓. In vitro: proliferation↓, migration↓, differentiation↓, secretion↓

Abbreviations: AERP, atrial refractory period; APD, action potential duration; CHF, congestive heart failure; CV, conduction velocity; HSP70, heat shock protein 70; IVST, interventricular septal thickness; JNK, c‐Jun N‐terminal kinase; LA, left atrial; LVPWT, LV posterior wall thickness; MMP‐9, matrix metalloprotein 9; NF‐κB, nuclear factor‐kappa B; P3NP, procollagen‐III N‐terminal propeptide; PPAR‐γ, peroxisome proliferator–activated receptor‐γ; STAT3, signal transducer and activator of transcription 3; TGF‐β, transforming growth factor‐β; TIMP, tissue inhibitor of matrix metalloproteinases; TLR4, Toll‐like receptor 4; TNF‐α, tumour necrosis factor alpha; TRPC3, transient receptor potential canonical‐3; α‐SMA, α‐smooth muscle actin.

## 6 AF ABLATION TARGETING ATRIAL FIBROSIS

Ablative methods have been designed to ameliorate catheter ablation via targeting of identified fibrotic areas, according to endocardial voltage mapping or heart MRI.^75^ Box isolation of fibrotic areas (BIFA) represents a novel promising individualized ablation protocol for AF management, targeting areas with pronounced fibrosis by circumferential isolation of left atrial fibrosis, which can effectively control the cardiac rhythm in cases of paroxysmal AF in spite of prolonged pulmonary vein (PV) isolation. This protocol was applied successfully for first AF ablation alongside PV isolation in non‐paroxysmal AF.^76^, ^77^, ^78^ Bachmann's bundle insertion ablation is another new method for achieving complete. This technique may contribute to the reduction in AF relapses.^78^, ^79^

## 7 CONCLUSION

Left atrial fibrosis is considered a potential major parameter in and predictor of AF treatment outcome. Atrial fibrosis represents an essential milestone in atrial function deterioration, causing atrial susceptibility to AF. Conversely, AF itself promotes atrial fibrosis, starting and sustaining the vicious cycle. LA fibrosis is also closely associated with thrombus generation and high stroke risk, particularly in AF patients. Therefore, improved understanding of pathophysiological mechanisms underpinning the early stages of atrial substrate development in AF could aid the identification of adequate imaging features for assessing the odds of new AF onset, suggesting pathways specifically targetable for prevention. In recent years, LGE‐MRI and LA strain detection allow for reproducible, non‐invasive assessment and follow‐up investigations to improve AF treatment in the clinic, which could increase clinical diagnostic criteria. Rate control, rhythm control and stroke prophylaxis are the cornerstones of AF therapy. Inhibitors of RAAS are recommended in AF treatment, in case arrhythmia is combined with additional myocardial fibrotic diseases. A wide range of potential drugs with anti‐fibrotic effects reduce atrial interstitial fibrosis and AF in experimental studies, albeit at various targets, including TGF‐β, inflammatory mediators and fibroblast‐associated proteins. However, further trials are required to properly evaluate the efficacies of all these drugs. Ablation strategy is applied by targeting detected areas of fibrosis, such as BIFA and Bachmann's bundle insertion ablation. This strategy requires confirmation and validation regarding effectiveness in multicentre prospective randomized trials and may provide a useful rhythm control concept in paroxysmal AF in spite of durable PV isolation.

## CONFLICT OF INTEREST

None declared.

## AUTHOR CONTRIBUTIONS

**Jin Ma:** Funding acquisition (equal); Writing‐original draft (equal); Writing‐review & editing (equal). **Qiuxiong Chen:** Funding acquisition (equal); Validation (equal); Writing‐review & editing (equal). **Shiyu Ma:** Conceptualization (equal); Funding acquisition (equal); Writing‐original draft (equal); Writing‐review & editing (equal).

## Data Availability

Author elects to not share data.
